# STAT3 inhibition suppresses proliferation of retinoblastoma through down-regulation of positive feedback loop of STAT3/miR-17-92 clusters

**DOI:** 10.18632/oncotarget.2546

**Published:** 2014-11-03

**Authors:** Dong Hyun Jo, Jin Hyoung Kim, Chang Sik Cho, Young-Lai Cho, Hyoung Oh Jun, Young Suk Yu, Jeong-Ki Min, Jeong Hun Kim

**Affiliations:** ^1^ Fight against Angiogenesis-Related Blindness (FARB) Laboratory, Clinical Research Institute, Seoul National University Hospital, Seoul, Republic of Korea; ^2^ Tumor Microenvironment Research Center, Global Core Research Center, Seoul National University, Seoul, Republic of Korea; ^3^ Department of Biomedical Sciences, College of Medicine, Seoul National University, Seoul, Republic of Korea; ^4^ Center for Nanosafety Metrology, Korea Research Institute of Standards and Science Daejeon, Republic of Korea; ^5^ Department of Ophthalmology, College of Medicine, Seoul National University, Seoul, Republic of Korea; ^6^ Research Center for Integrated Cellulomics, Korea Research Institute of Bioscience and Biotechnology, Daejeon, Republic of Korea

**Keywords:** Retinoblastoma, STAT3 transcription factor, miR-17-92

## Abstract

Retinoblastoma, the most common intraocular malignant tumor in children, is characterized by the loss of both functional alleles of *RB1* gene, which however alone cannot maintain malignant characteristics of retinoblastoma cells. Nevertheless, the investigation of other molecular aberrations such as matrix metalloproteinases (MMPs) and miRNAs is still lacking. In this study, we demonstrate that STAT3 is activated in retinoblastoma cells, Ki67-positive areas of *in vivo* orthotopic tumors in BALB/c nude mice, and human retinoblastoma tissues of the advanced stage. Furthermore, target genes of STAT3 including *BCL2, BCL2L1, BIRC5,* and *MMP9* are up-regulated in retinoblastoma cells compared to other retinal constituent cells. Interestingly, STAT3 inhibition by targeted siRNA suppresses the proliferation of retinoblastoma cells and the formation of *in vivo* orthotopic tumors. In line with these results, STAT3 siRNA effectively induces down-regulation of target genes of STAT3. In addition, miRNA microarray analysis and further real-time PCR experiments with STAT3 siRNA treatment show that STAT3 activation is related to the up-regulation of miR-17-92 clusters in retinoblastoma cells via positive feedback loop between them. In conclusion, we suggest that STAT3 inhibition could be a potential therapeutic approach in retinoblastoma through the suppression of tumor proliferation.

## INTRODUCTION

Various molecular events lead to initiation and progression of malignant tumors. As for retinoblastoma, the most common intraocular malignancy in children, the loss of both functional alleles of retinoblastoma gene (*RB1* gene) is known to initiate most cases [1–3]. Then, other genetic aberrations are involved in the progression of retinoblastoma [4, 5]. In addition, epigenetic regulation, matrix metalloproteinases (MMPs), and non-conding RNAs such as miRNAs provide additional layers of complexity in the pathogenesis of retinoblastoma [6–10]. For example, MMP9 is up-regulated in the proliferation of retinoblastoma cells [6] and is highly expressed in tumor tissues with optic nerve invasion [7]. Similarly, there is increasing evidence for the role of miRNAs in the progression of retinoblastoma [8]. In particular, *Rb*/*p107* double knockout mice and human retinoblastoma patients demonstrate high expression of miR-17-92 clusters in tumor tissues [9] and the inactivation of miR-17-92 by synthetic lethality suppresses the formation of retinoblastoma in mice [10]. These studies definitely give insight for the development of therapeutic approaches against retinoblastoma besides currently available chemotherapeutic agents including vincristine, carboplatin, and etoposide [11]. However, clinical use of molecularly targeted therapy against retinoblastoma is yet to be widely implanted.

It is reasonable to find out potential therapeutic targets which modulate and reinforce aforementioned molecular characteristics of retinoblastoma other than *RB1* gene mutation. STAT3 can be an excellent example. A notable fact is that STAT3 is constitutively activated in 70% of solid cancers [12]. Furthermore, as a transcription factor, activated STAT3 is involved in various cellular functions by up-regulation of target genes including *CCND1, CDKN1A* (cell cycle), *BCL2, BCL2L1, BIRC5, MYC* (apoptosis-related), *MMP9* (migration, invasion), and *VEGF* (angiogenesis) [13]. Accordingly, STAT3 is recognized as a signaling hub or a central mediator of cellular events in malignant cells [13–15]. In this context, STAT3 inhibition might be an effective approach in the treatment of cancers in which STAT3 is aberrantly activated.

In this study, we demonstrated that STAT3 was activated in retinoblastoma tissues from human patients. This phenomenon was also observed in retinoblastoma cells and *in vivo* orthotopic tumors. In line with these results, we showed increased expression of *BCL2, BCL2L1, BIRC5,* and *MMP9* genes, target genes of STAT3, in retinoblastoma cells compared to other retinal constituent cells, retinal pigment epithelial cells and retinal endothelial cells. Furthermore, inhibition of STAT3 in retinoblastoma cells with targeted siRNAs resulted in impaired proliferation and down-regulation of target genes. We also demonstrated that STAT3 inhibition suppressed formation and proliferation of *in vivo* orthotopic tumors. In addition, we showed that the action of STAT3 in retinoblastoma was linked to miR-17-92 clusters, which acted as oncogenic miRNAs, via positive feedback loop between them. Taken together, our results suggested that STAT3 inhibition have a therapeutic potential against retinoblastoma through the suppression of tumor proliferation.

## RESULTS

### STAT3 is activated in retinoblastoma tissues from human patients, retinoblastoma cells, and *in vivo* orthotopic tumors

**Figure 1:**
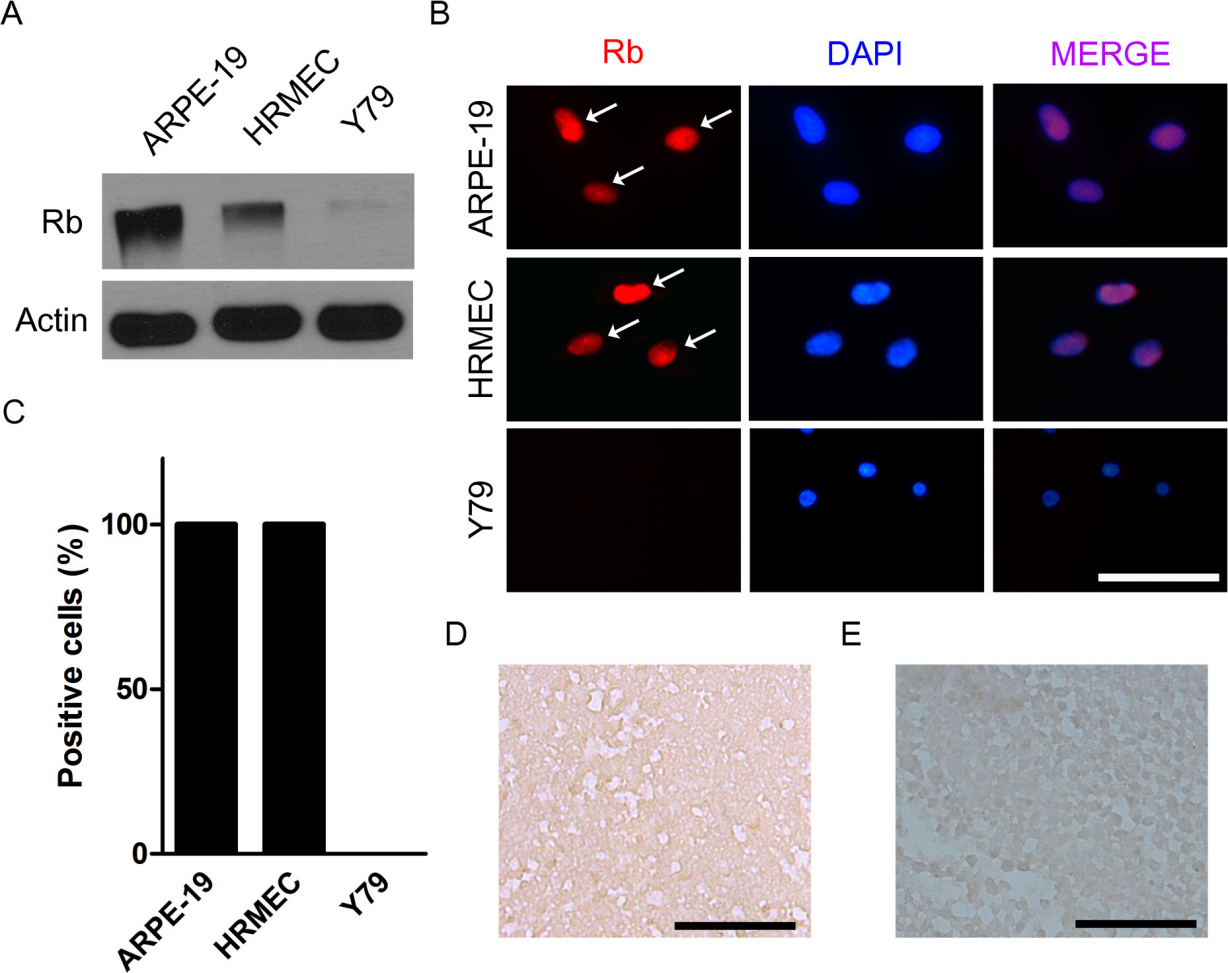
Retinoblastoma is characterized by the loss of functional alleles of *RB1* gene **(A)** Expression of retinoblastoma protein in ARPE-19 cells, HRMECs, and Y79 cells. **(B)** ARPE-19 cells, HRMECs, and Y79 cells were stained with the antibody to retinoblastoma protein. White arrows indicate positively stained nuclei. **(C)** Quantitative analysis of immunocytochemical staining. **(D)** A representative example of the expression of retinoblastoma protein analyzed by immunohistochemical staining in *in vivo* orthotopic tumors at 4 weeks after intravitreal injection of Y79 cells. **(E)** A representative example of the expression of retinoblastoma protein in human retinoblastoma tissues from a 21-month-old male patient. All scale bars represent 50 μm. Rb, retinoblastoma protein.

Most cases of retinoblastoma are characterized by the loss of both functional alleles of *RB1* gene in tumors [1, 2]. Y79 cells, one of the most widely utilized retinoblastoma cell lines, are also known to be negative for *RB1* gene. Western blot and immunocytochemical staining showed that there was no evidence of retinoblastoma protein in Y79 cells. In contrast, all ARPE-19 cells and human retinal microvascular endothelial cells (HRMECs) demonstrated definite nuclear staining of retinoblastoma protein (Fig. 1A-C). Similar to *in vitro* immunoblot and immunocytochemical analysis, immunohistochemical staining evidenced that there was no expression of retinoblastoma protein in *in vivo* orthotopic tumors (Fig. 1D). Furthermore, we also observed that retinoblastoma tissues from 6 patients in this study did not demonstrate positive staining of retinoblastoma protein in tumor cells which could be shown in normal retinal tissues (Fig. 1E and Supplemental Table 1).

Next, we planned to figure out other aberrations in retinoblastoma cells which played a role in modulation of various cellular events regarding the progression of retinoblastoma. Antibody array with 269 well-characterized antibodies against proteins associated with cancer signaling demonstrated that various proteins were differentially expressed in Y79 cells compared to HRMECs (full raw data of antibody array were provided as Supplemental Information). Among top 5 up-regulated phosphorylated proteins in Y79 cells to HRMECs, we pinpointed STAT3 for further investigation (Supplemental Table 2). STAT3 is a well-known signaling hub to mediate various cancer-related cellular functions including cell cycle, anti-apoptosis, migration, invasion, and angiogenesis [13, 15]. Furthermore, retinoblastoma protein negatively regulates the transcriptional activity of STAT3 by directly binding to it [16]. In this context, it might be plausible for *RB1*-negative retinoblastoma cells to show constitutive activation of STAT3.

**Figure 2:**
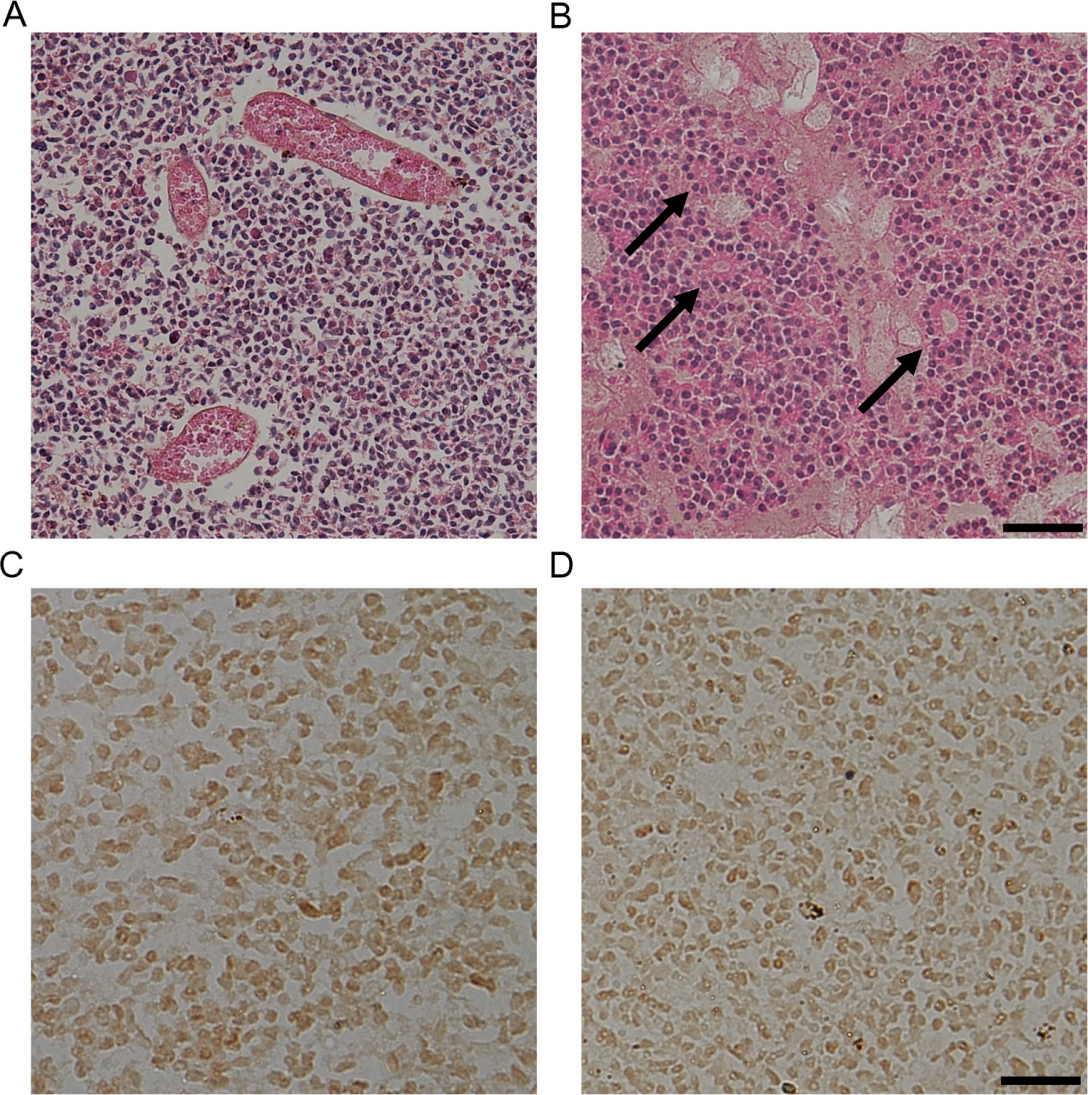
STAT3 is activated in human retinoblastoma tissues **(A-B)** Representative examples of the areas with proliferating cells and tumor vessels (A) and rosettes (arrows, B) in human retinoblastoma tissues. Scale bar represents 100 μm. **(C-D)** Representative examples of the expression of pSTAT3^Ser727^ (C) and pSTAT3^Tyr705^ (D) in human retinoblastoma tissues (*n* = 6). Scale bar represents 50 μm.

To investigate the expression of pSTAT3^Ser727^ and pSTAT3^Tyr705^ in human retinoblastoma tissues, we performed immunohistochemical staining with retinoblastoma tissues from 6 patients who underwent enucleation in a month after the diagnosis. All tumors were diagnosed as class Va in Reese-Ellsworth classification and class E according to the International Classification of Retinoblastoma classification (Supplemental Table 1). Retinoblastoma tissues are characterized by heterogenous histologic patterns even in same tumors: some regions are filled with tumor cells and abundant tumor vasculatures (Fig. 2A) and others demonstrate distinctive rosette formation (Fig. 2B). In advanced stage, all tumor tissues in this study demonstrated highly positive nuclear staining of pSTAT3^Ser727^ and pSTAT3^Tyr705^ regardless of histological patterns (Fig. 2C, D).

**Figure 3:**
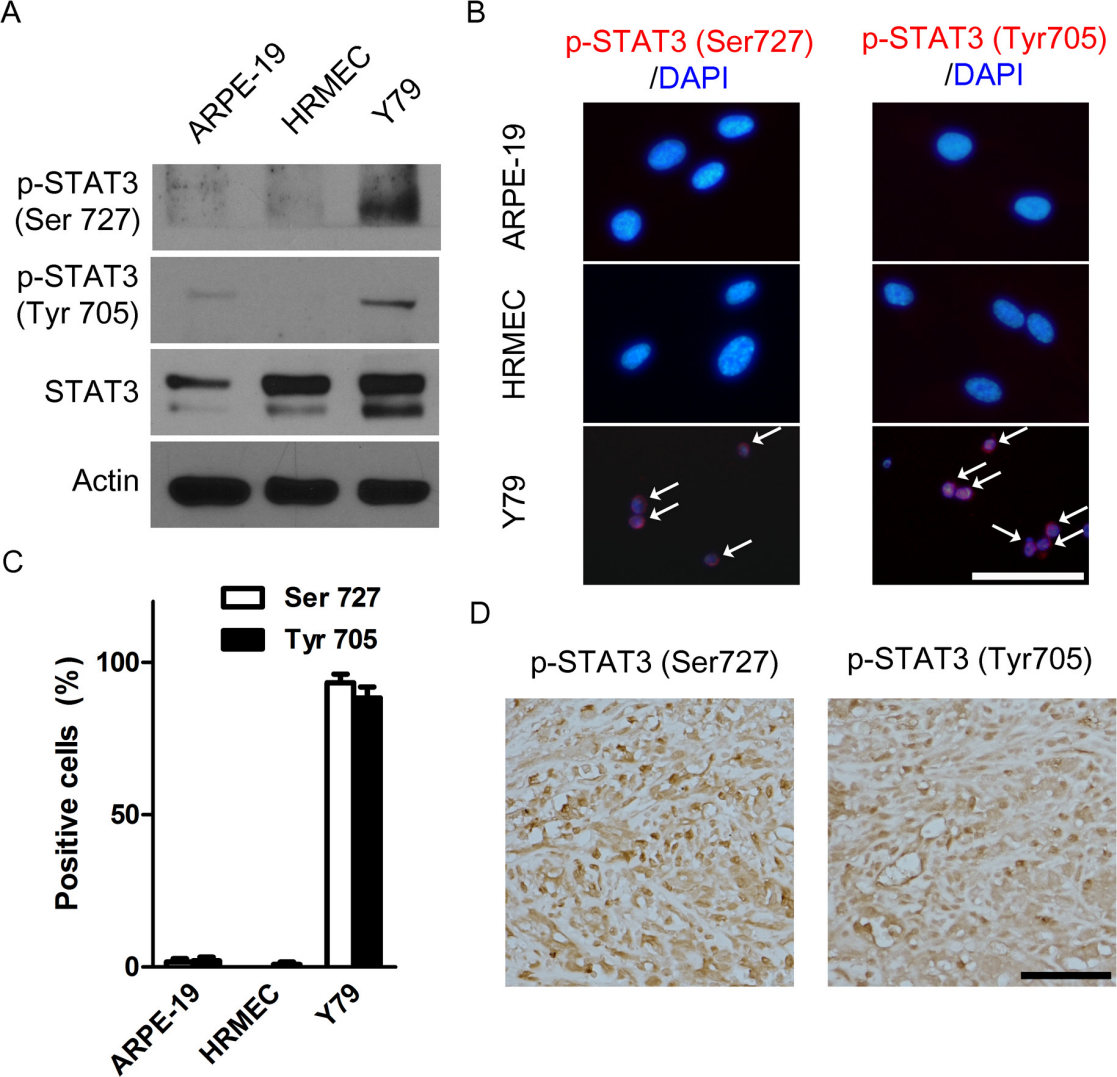
STAT3 is activated in retinoblastoma cells and *in vivo* orthotopic tumors **(A)** pSTAT3^Ser727^ and pSTAT3^Tyr705^ in whole cell extracts of ARPE-19 cells, HRMECs, and Y79 cells. **(B)** Representative images of immunocytochemical staining of pSTAT3^Ser727^ and pSTAT3^Tyr705^ in ARPE-19 cells, HRMECs, and Y79 cells. White arrows indicate positively stained nuclei. **(C)** Quantitative analysis of immunocytochemical staining. **(D)** A representative example of the expression of pSTAT3^Ser727^ and pSTAT3^Tyr705^ in Ki-67 positive areas of *in vivo* orthotopic tumors at 4 weeks after intravitreal injection of Y79 cells (*n* = 6). All scale bars represent 50 μm.

Similarly to antibody array data, Y79 cells demonstrated higher expression of both pSTAT3^Ser727^ and pSTAT3^Tyr705^ than ARPE-19 cells and HRMECs (Fig. 3A and Supplemental Fig. 1). Interestingly, 93.3% and 88.3% of Y79 cells were positive for pSTAT3^Ser727^ and pSTAT3^Tyr705^, respectively (Fig. 3B, C). To figure out whether this *in vitro* expression was maintained during *in vivo* proliferation of tumor cells, we evaluated the expression of activated forms of STAT3 in *in vivo* orthotopic tumors. In particular, we observed dense nuclear staining of pSTAT3^Ser727^ and pSTAT3^Tyr705^ in areas of positive for Ki67 (where more than 80% of cells showed positivity), a proliferation marker (Fig. 3D). These results might imply the relation between STAT3 activation and proliferation of retinoblastoma cells.

### Target genes of STAT3 are differentially expressed in retinoblastoma cells compared to other retinal constituent cells

**Figure 4:**
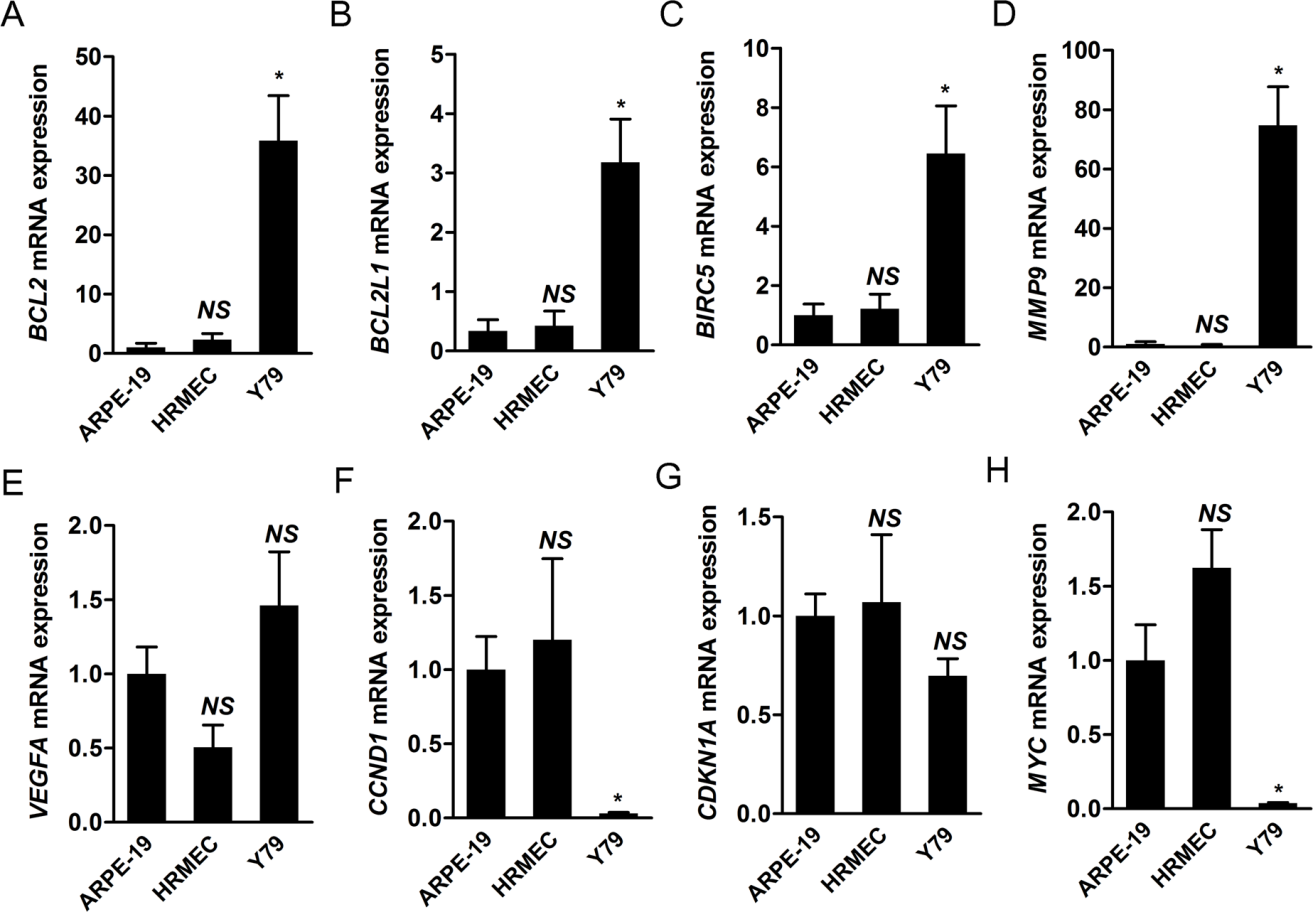
Target genes of STAT3 are differentially expressed in retinoblastoma cells compared to other retinal constituent cells **(A-H)** We evaluated the relative levels of mRNA expression of various target genes of STAT3 from ARPE-19 cells, HRMECS, and Y79 cells under normal culture conditions through real-time PCR analysis. A, *BCL2*. B, *BCL2L1*. C, *BIRC5*. D, *MMP9*. E, *VEGFA*. F, *CCND1*. G, *CDKN1A*. H, *MYC*. *NS*, not significant (*P* > 0.05); *P < 0.05.

Then, we evaluated the expression of target genes of STAT3 in Y79 cells compared to that in ARPE-19 cells and HRMECs under normal culture conditions. Among target genes of STAT3, we chose a representative set of genes according to their known functions: *BCL2, BCL2L1, BIRC5, MYC* (apoptosis-related), *MMP9* (migration, invasion), *CCND1, CDKN1A* (cell cycle), and *VEGF* (angiogenesis). Interestingly, mRNA expression of *BCL2, BCL2L1, BIRC5,* and *MMP9* was significantly higher in Y79 cells than other retinal constituent cells (*P* < 0.05; Fig. 4A-D). A notable fact is that MMP9, which demonstrated the most up-regulated mRNA expression, reflected the level of proliferation [6] and invasiveness of retinoblastoma cells [7]. STAT3 activation might be involved in tumor progression by modulation of the expression of factors related with cancer such as MMP9.

In contrast, *VEGFA, CCND1, CDKN1A*, and *MYC* were not up-regulated in Y79 cells (Fig. 4E-H). We speculated that the differential expression of target genes of STAT3 might be due to influences from the network including other molecular characteristics such as non-coding RNAs and specific gene amplification including *MYCN* in Y79 cells. As for *CCND1* and *CDKN1A*, 3′ untranslated regions (UTRs) of both genes have sequences aligned with some of miR-17-92 clusters which is elevated in retinoblastoma [9, 10]. Database search in microRNA.org demonstrated that sequences from 998 through 1020 in the 3′ UTR region of *CCND1* are targets for miR-17 and miR-20a, those from 1770 through 1784 are those for miR-19a, and those from 1777 through 1782 are those for miR-19b. Similarly, sequences from 461 through 474, from 1133 through 1154, and from 1138 through 1154 in the 3′ UTR region of *CDKN1A* are targets for miR-17 and miR-20a. The influences of miR-17-92 clusters on retinoblastoma cells might impede the effects of STAT3 activation on these genes. We speculated that down-regulation of *MYC* was related with N-myc amplification in Y79 cells as in neuroblastoma [17]. The results in *VEGFA* expression might be due to the fact that retinal pigment epithelial cells are good sources of VEGF for neighboring cells in the retina [18].

### STAT3 inhibition via targeted siRNA induces impaired proliferation of retinoblastoma cells and down-regulation of target genes of STAT3

**Figure 5:**
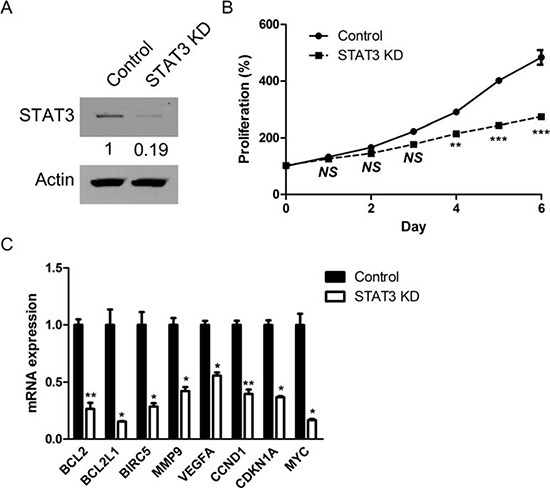
STAT3 inhibition via targeted siRNA induces impaired proliferation of retinoblastoma cells and down-regulation of target genes of STAT3 **(A)** The expression of STAT3 protein in Y79 cells after the treatment with siRNA targeting to STAT3. **(B)** The relative levels of proliferation of Y79 cells according to the treatment with scramble siRNA (Control) and STAT3 siRNA (STAT3 KD). **(C)** Quantitative analysis of relative mRNA expression of target genes of STAT3 according to the treatment with scramble siRNA (Control) and STAT3 siRNA (STAT3 KD). *NS*, not significant (*P* > 0.05); *P < 0.05; **P < 0.01; ***P < 0.001.

To investigate the roles of STAT3 in the proliferation of retinoblastoma cells with differential effects on the expression of representative target genes, we down-regulated the expression of STAT3 by the treatment with siRNA targeted to *STAT3* (Fig. 5A and Supplemental Fig. 2). Interestingly, STAT3 siRNA suppressed the proliferation of Y79 cells compared to scramble siRNA (Fig. 5B). Furthermore, the expression of various target genes of STAT3, regardless of the differential expression patterns in Y79 cells, was also effectively down-regulated by the treatment with STAT3 siRNA (Fig. 5C). We confirmed the data of siRNA transfection with another STAT3-targeting siRNA with different sequences (Supplemental Table 4 and Supplemental Fig. 3, 4).

### Intravitreal administration of STAT3 siRNA suppresses *in vivo* formation of orthotopic tumors

**Figure 6:**
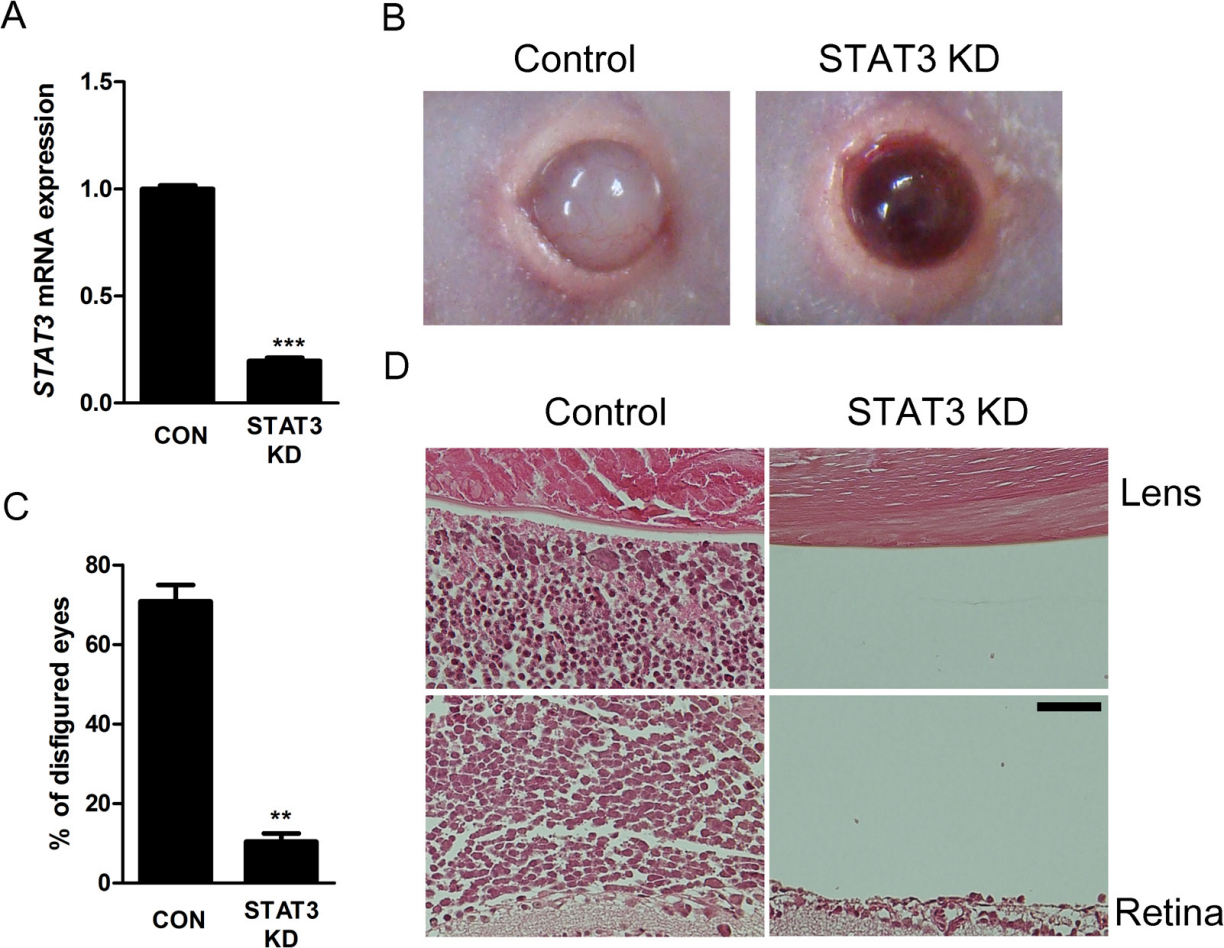
Intravitreal administration of STAT3 siRNA suppresses *in vivo* formation of orthotopic tumors **(A)** The mRNA expression of *STAT3* gene in *in vivo* orthotopic tumors according to intravitreal administration of scramble siRNA or STAT3 siRNA (*n* = 6 - 8, 3 replicates). **(B)** Representative external photographs of eyes of mice according to intravitreal administration of scramble siRNA or STAT3 siRNA. **(C)** Quantitative analysis of disfigured eyes according to intravitreal administration of scramble siRNA or STAT3 siRNA (*n* = 6 - 8, 3 replicates). **(D)** Representative examples of H&E staining of *in vivo* orthotopic tumors. CON or Control, scramble siRNA treatment. STAT3 KD, STAT3 siRNA treatment. Scale bar represents 50 μm. **P < 0.01; ***P < 0.001.

To further investigate the roles of STAT3 in *in vivo* proliferation of retinoblastoma, we planned to test the therapeutic potential of STAT3 inhibition in *in vivo* orthotopic tumors. At 2 weeks after the injection of Y79 cells into the vitreous cavity, we intravitreally injected 1 μL of scramble siRNA or STAT3 targeting siRNA (100 nM). Interestingly, the treatment effectively suppressed the expression of *STAT3* mRNA (*P* < 0.001, Fig. 6A). Furthermore, STAT3 siRNA decreased the proportion of the formation of externally visible tumors (*P* < 0.01, Fig. 6B, C). Histologic examination showed that there was much less evidence of tumor cells in the vitreous cavity between the lens and the retina in mice receiving STAT3 siRNA treatment, compared to the eyes which were full of tumors in control mice (Fig. 6D).

### Effects of STAT3 activation on retinoblastoma cells are regulated by positive feedback loop of STAT3/miR-17-92 clusters

**Figure 7:**
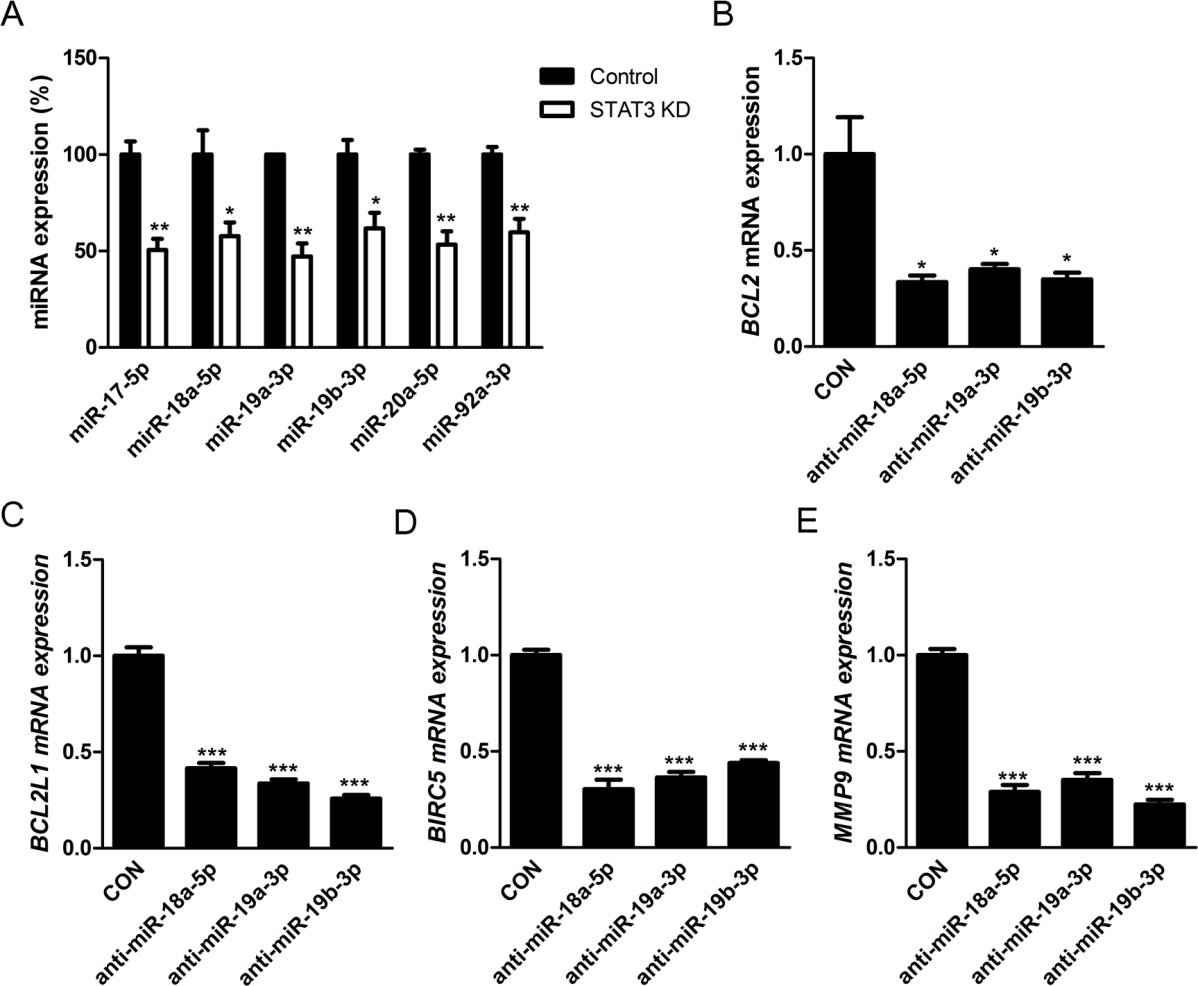
Effects of STAT3 activation on retinoblastoma cells are regulated by positive feedback loop of STAT3/miR-17-92 clusters **(A)** The relative expression of miR-17-92 clusters according to according to the treatment with scramble siRNA (Control) or STAT3 siRNA (STAT3 KD). **(B-E)** Quantitative analysis of the relative levels of the expression of various target genes of STAT3 according to the treatment with scramble inhibitor (CON) or specific inhibitors to miRNAs of miR-17-92 clusters. B, *BCL2*. C, *BCL2L1*. D, *BIRC5*. E, *MMP9*. *P < 0.05; **P < 0.01; ***P < 0.001.

Next, we investigated the roles of STAT3 activation on the expression of miR-17-92 clusters, which are highly expressed in retinoblastoma cells in our previous study and other groups' studies on miRNA expression [9, 10, 19]. To confirm these results, we performed miRNA microarray analysis with Y79 cells, evidencing increased clustered expression of miR-17-92 clusters (Supplemental Table 6). As a transcription factor, STAT3 binds to a highly conserved site in the promoter region of C13orf25, which encodes miR-17-92 clusters, as well as the promoter regions of other target genes [20]. Real-time PCR analysis demonstrated that STAT3 siRNA down-regulated the expression of miR-17-92 clusters in retinoblastoma cells as a whole compared to scramble siRNA (Fig. 7A).

In order to figure out feedback effects of miR-17-92 clusters on STAT3 activation, we evaluated the expression of target genes of STAT3 which demonstrated higher expression in Y79 cells than other retinal constituent cells: *BCL2, BCL2L1, BIRC5,* and *MMP9* according to the treatment with specific miRNA inhibitors to components of miR-17-92 clusters: miR-18a-5p, miR-19a-3p, and miR-19b-3p. This set of miRNAs was deliberately chosen based on the screening data showing that there were no definite differences in the positivity of immunostaining of pSTAT3^Ser727^ and pSTAT3^Tyr705^ after the treatment with miR inhibitors to miR-17-5p, miR-20a-5p, and miR-92a-3p (Supplemental Fig. 5). Interestingly, miRNA inhibitors targeted to miR-18a-5p, miR-19a-3p, and miR-19b-3p down-regulated the expression of *BCL2, BCL2L1, BIRC5,* and *MMP9*, target genes of STAT3, which implied the positive feedback loop of STAT3/miR-17-92 clusters (Fig. 7B-E). In particular, inhibition of miR-18a-5p, miR-19a-3p, and miR-19b-3p resulted in differential up-regulation of mRNA expressioin of *PIAS3, SOCS1,* and *SOCS3*, coding genes for regulatory proteins of STAT3 such as (Supplemental Fig. 6–8). In this way, the expression of these miRNAs affected the amount of activated STAT3, and vice versa.

## DISCUSSION

This study showed that multifunctional roles of STAT3 activation in the proliferation of retinoblastoma cells as a main regulator of the expression of various target genes and miR-17-92 clusters and the therapeutic potential of STAT3 inhibition in retinoblastoma. Interestingly, down-regulation of some components of miR-17-92 clusters resulted in decreased expression of target genes of STAT3, implying that they also affected the action of STAT3, composing the positive feedback loop. We speculated that these results might provide a novel therapeutic approach targeting to STAT3 in the treatment of retinoblastoma. Currently, most of chemotherapeutic agents against retinoblastoma are targeting to cytoskeleton structures or nucleic acid-topoisomerase complexes [11]. This trend mirrors the main initiating factor in the pathogenesis of retinoblastoma: loss of functional alleles of *RB1* gene and resultant inactive retinoblastoma protein, which is involved in the control of cell cycle [1, 2]. In addition, although there is much evidence on other molecular aberrations regarding the progression of retinoblastoma [1, 4, 5, 8], the overall mutational rate in retinoblastoma patients is known to be quite low [21]. In this context, molecularly targeted therapy is yet to be implanted in clinical settings for retinoblastoma.

Nevertheless, we should focus on novel therapeutic approaches targeting to other molecular aberrations which might have roles in the progression of retinoblastoma, because *Rb1* mutation is not a sufficient condition for the malignant characteristics of retinoblastoma cells [22, 23]. Although the loss of functional *Rb1* gene is one of the hallmarks of retinoblastoma [1], its major effect is the initiation of stable retinoma, a benign tumor, with low level genomic instability [22]. In addition, retinal precursors without retinoblastoma protein demonstrates high proliferative capacity but require additional anti-apoptotic mutation to be intrinsically death-resistant [23]. These reports implied that other molecular factors might play an important role in the malignant progression of retinoblastoma. In this context, we screened the expression of cancer-related proteins with antibody array techniques in retinoblastoma cells compared to HRMECs, Among the proteins which demonstrated higher phosphorylation in Y79 cells than HRMECs, we focused on STAT3, because it modulates the expression of various target genes and miRNAs, acting as a signaling hub and main regulator of cellular events regarding carcinogenesis [12, 13].

A widely accepted concept on STAT3 activation is that there is a sequence of phosphorylation of STAT3 at different residues [24, 25]. In general, phosphorylation of STAT3 at Tyr705 leads to homodimerization of STAT3 by binding between this residue and SH2 domain of the counterpart STAT3. Then, transcription activation is induced by further phosphorylation of STAT3 at Ser727. Nevertheless, because there is a concern on independent or inverse phosphorylation of STAT3 at Tyr705 and Ser727 [26], it is important to investigate thoroughly on the activation of STAT3 at different residues. Furthermore, acetylation is also a critical regulator of STAT3 with posttranslational modification [27].

Our results demonstrated that STAT3 activation induced up-regulation of *BCL2, BCL2L1, BIRC5,* and *MMP9* genes in retinoblastoma cells. The former 3 genes are known to be related with anti-apoptosis [13]. In this context, the treatment with STAT3 siRNA suppressed *in vitro* proliferation of retinoblastoma cells. These *in vitro* results corresponded to observations in *in vivo* orthotopic tumors that showed relations between positivity of pSTAT3 and Ki67, a proliferation marker. Furthermore, advanced human retinoblastoma in this study also demonstrated highly positive nuclear staining of pSTAT3 in immunohistochemical analysis. We speculated that STAT3 activation in retinoblastoma cells induced proliferation of retinoblastoma cells by up-regulation of target genes with anti-apoptotic activity. In addition, MMP9 reflected the proliferation of retinoblastoma cells [6] and was highly positive in the tumors with optic nerve invasion, which meant invasiveness of retinoblastoma [7]. Although there was no definite evidence of optic nerve invasion in our cases, we could easily assume that advanced stage of our cases (all cases were graded as Va in Reese-Ellsworth Classification and E in International Classification of Retinoblastoma) might be related with STAT3 activation.

Another implication of STAT3 activation in retinoblastoma in this study was the overexpression of the well-known oncogenic miRNA family, miR-17-92 clusters. As previously mentioned, *RB1* mutation alone does not induce malignant transformation of tumor cells [22, 23]. Interestingly, miR-17-92 clusters are highly expressed in human retinoblastoma [10, 28]. Furthermore, over-expression of miR-17-92 clusters with deletion of *Rb1* and *Rbl1* accelerated the emergence of retinoblastoma with frequent metastasis to the brain in mice [9]. In the same context, miR-17-92 inactivation suppressed retinoblastoma formation in mice and could be a potential therapeutic approach in retinoblastoma [10, 28]. Our data demonstrated that STAT3 activation was related with the over-expression of miR-17-92 clusters in retinoblastoma cells. The mechanism might be the binding of STAT3 on the promoter region of miR-17-92 gene just as that of various target genes of STAT3 [20].

The more interesting part of the relation between STAT3 and miR-17-92 clusters in retinoblastoma cells was the positive feedback. Interestingly, the inhibition of miR-18a-5p, miR-19-3p, and miR-19b-3p induced the decrease in the proportion of pSTAT3-positive retinoblastoma cells. Likewise, the treatment suppressed the transcription of target genes of STAT3 including *BCL2, BCL2L1, BIRC5,* and *MMP9* which demonstrated higher expression in Y79 cells than other retinal constituent cells, ARPE-19 cells and HRMECs. These results implied that the positive feedback loop between activated STAT3 and miR-17-92 governed the expression of them. Based on the experiments using specific miR inhibitors, the mechanisms of the effects of miR-18a-5p, miR-19a-3p, and miR-19b-3p on the up-regulation of activated STAT3 might be the suppression of genes for regulatory proteins of STAT3 such as protein inhibitor of activated STAT3 (*PIAS3*) and suppressor of cytokine signaling 1 and 3 (*SOCS1, SOCS3*) [29–32].

In summary, we found that STAT3 activation in retinoblastoma mediated various cellular events including the expression of genes related with anti-apoptotic activity and migration/invasion and the up-regulation of oncogenic miRNA, miR-17-92 clusters. In particular, the positive feedback loop of STAT3/miR-17-92 clusters might reinforce the activation of these signals in retinoblastoma. In line with these results, STAT3 inhibition by targeted siRNA suppressed the proliferation of retinoblastoma cells, decreased the expression of target genes, and effectively inhibited the formation of *in vivo* orthotopic tumors. Based on our data, we suggested that STAT3 inhibition could be a potential therapeutic approach in retinoblastoma beyond the focus on aberrant cell cycle machineries related with *RB1* gene mutation.

## METHODS

### Primary antibodies

The list of primary antibodies was as follows: pSTAT3^Ser727^ (host: rabbit, cat. no. 9134, Cell Signaling), pSTAT3^Tyr705^ (host: mouse, cat. no. 9138, Cell Signaling), retinoblastoma protein (host: mouse, cat. no. 9309, Cell Signaling), and β-actin (host: rabbit, cat. no. A2066, Sigma).

### Immunohistochemistry

Sagittal sections were performed at 4 μm thickness from paraffin blocks of enucleated eyes from *in vivo* tumor models (n = 6) and human patients (n = 6). Immunohistochemical analysis with human eyes was approved by Institutional Review Board of Seoul National University Hospital (H-1404-032-568) and clinical demographics of 6 patients were summarized in Supplemental Table 1. We also followed Declaration of Helsinki during the whole procedures with human tissue samples. The sections were incubated at 60°C for 2 hours and processed with deparaffinization and hydrated by sequential immersion in Xylene Substitute (Thermo) and graded ethyl alcohol solutions. Antigen retrieval was performed by the treatment with 0.1 M sodium citrate (pH 6.8, Sigma) at 120°C for 10 minutes. The sections were permeabilized with 0.2% Triton X-100 at room temperature for 10 minutes. Then, we treated the sections with 1X Universal Blocking Reagent (Biogenex) for 10 minutes to minimize nonspecific binding. After incubation with primary antibodies (1:250) overnight, the sections were treated with REAL™ Detection Systems (Dako) and DAB Kit (Life Technologies) as the manufacturer's instructions. Then, the slides were mounted with Permount solution (Thermo) and observed under the light microscope (Nikon).

### Cells

ARPE-19 cells (ATCC), HRMECs (ACBRI), Y79 cells (ATCC) were maintained in normal culture conditions. Specific culture conditions were as follows: DMEM (Gibco) with 10% FBS (Gibco) for ARPE-19, EBM-2 (Lonza) with SingleQuot Kit Suppl. & Growth Factors (Lonza) for HRMECs, and RPMI-1640 (Thermo) with 10% FBS (Gibco) for Y79 cells.

### Western blot analysis

Equal amount of extracted proteins (50 μg) from whole cell lysates of ARPE-19 cells, HRMECs, and Y79 cells was separated by 7% SDS-PAGE and transferred to a nitrocellulose membrane (GE). After overnight incubation with primary antibodies (1:1,000 for pSTAT3^Ser727^, pSTAT3^Tyr705^, retinoblastoma protein and 1:5,000 for β-actin) at 4°C, the membranes were incubated with species-specific peroxidase-conjugated secondary antibodies (Thermo) for 1 hour at room temperature. Then, they were treated with EZ-Western detection kit (Daeillab) and exposed to the film. Exposed films were scanned using the scanner.

### Immunocytochemistry

Cells were plated on a 6-well plate (for Y79 cells, the well is coated with 0.1 mg/mL poly-D-lysine) and incubated for 24 hours at 37°C. Then, they were fixed with 4% paraformaldehyde for 15 minutes. After permeabilization with 0.1% Triton X-100 for 15 minutes, we treated the cells with 3% bovine serum albumin (BSA) for 2 hours to minimize nonspecific binding. Then, the cells were incubated with primary antibodies in 3% BSA (1:250) overnight at 4°C. Next day, we treated the cells with species-specific Alexa Flour® 594 Goat IgG Antibody (Life Technologies) in 3% BSA (1:1,000) for 1 hour. Nuclear staining was performed with 4′, 6-diamidino-2-phenylindole dihydrochloride (Sigma) staining. Then, the cells were observed with a fluorescence microscope (Leica).

### Mice

6-week-old male BALB/c nude mice were purchased from Central Lab. Animal and maintained under a 12-hour dark-light cycle. We followed an ARVO statement for the use of animals in ophthalmic and vision research and the guidelines by Seoul National University Institutional Animal Care and Use Committee in the care, use, and treatment of all animals.

### *In vivo* orthotopic transplantation

We developed *in vivo* orthotopic tumor model with Y79 cells as previously described [33]. Briefly, Y79 cells were prepared in serum-free RPMI-1640 at the concentration of 1 × 10^5^ cells/μL. After adequate anesthesia, we injected cell suspension (1 μL) into the vitreous cavity of the right eyes of mice. For immunohistochemistry, mice (n = 6) were sacrificed with CO_2_ inhalation and enucleated eye were prepared for paraffin blocks at 4 weeks after the injection of tumor cells.

### Real-time PCR

Total RNA from cell lysates was isolated using TRI Reagent (Molecular Research Center) and cDNA was prepared with High Capacity RNA-to-cDNA Kit (Life Technologies). For miRNA analysis, reverse transcription was performed using TaqMan® MicroRNA Reverse Transcription Kit (Life Technologies) and miRNA pools were isolated using mirVana™ miRNA Isolation Kit (Life Technologies) as the manufacturer's instructions. Real-time PCR was performed with StepOnePlus Real-Time PCR System (Life Technologies) using TaqMan® Fast Advanced Master Mix (Life Technologies) with Gene Expression Assays (Life Technologies) for gene expression analysis and TaqMan® Universal Master Mix II (Life Technologies) with miRNA assays (Life Technologies) for miRNA analysis. Specific primers for gene expression and miRNA analysis were provided in Supplenentary Table 3. All procedures were performed in accordance with the MIQE guidelines.

### miRNA target analysis using web-based database

To analyze the results of differences in mRNA expression of various target genes in different cell lines and in Y79 cells according to the treatment with miRNA inhibitors, we searched through web-based database provided by microRNA.org (http://www.microrna.org/microrna/home.do) [34]. Briefly, we inserted the gene names of target genes of STAT3 into ‘Target mRNA’ and Homo sapiens into ‘Species’. Then, we could get plausible alignment details of binding between miRNA and 3′ UTR of target genes. Only target sites of conserved miRNAs with good mirSVR scores were taken into consideration in the analysis.

### Treatment of siRNA or miRNA inhibitors

Y79 cells were plated on 100-mm dish coated with 0.1 mg/mL poly-D-lysine. After 24 hours of incubation, cells were treated with Lipofectamine® RNAiMAX Transfection Reagent (3 μL per 1 mL media, Life Technologies) and siRNAs (100 nM) or miRNA inhibitors (30 nM) in Opti-MEM® I Reduced Serum Medium (Life Technologies). Specific siRNA sequences and miRNA inhibitors were provided in Supplemental Table 4 and 5, respectively. At 24 hours after the transfection, the cells were prepared for further gene expression or miRNA analysis. To investigate the effects of STAT3 siRNA treatment on proliferation of cells, the cells were counted daily at randomly selected 10 fields in each dish at × 200 magnification.

### Intravitreal injection of siRNA

For the evaluation of therapeutic efficacy of STAT3 siRNA (siRNA no. 1145658 and 1145659, Bioneer), we prepared siRNA in 1X siRNA buffer (GE) at the concentration of 100 nM. Then, we injected suspension of negative scramble siRNA or STAT3 siRNA (1 μL) into the vitreous cavity of the right eye of mice at 2 weeks after the injection of tumor cells. After additional 2 weeks, the mice were sacrificed with CO_2_ inhalation and enucleated eyes were prepared for H&E staining.

### miRNA microarray

Total RNA from cell lysates of Y79 cells was isolated using TRI Reagent (Molecular Research Center) and delivered to eBiogen for further analysis. The synthesis of target miRNA probes and hybridization were performed using Agilent's miRNA Complete Labeling and Hyb Kit. Dephosphorylated RNAs were ligated with pCp-Cy3 mononucleotide and purified with MicroBioSpin 6 columns (Bio-Rad). After purification, labeled samples were suspended with Gene Expression Blocking Agent (Agilent) and Hi-RPM Hybridization Buffer (Agilent). Then, denatured labeled probes were pipetted onto assembled Agilent Human miRNA Microarray (8 × 60K) and hybridized for 20 hours at 55°C in Microarray Hybridization Oven (Agilent). The hybridized images were scanned using DNA Microarray Scanner (Agilent) and quantified with Feature Extaction Software (Agilent). Normalization was performed with GeneSpringGX 7.3 (Agilent) by data transformation (set measurements less than 0.01 to 0.01) and per chip normalization (normalized to 75^th^ percentile). The data discussed in this publication have been deposited in NCBI's Gene Expression Omnibus [35] and are accessible through GEO series accession number GSE58092 (http://www.ncbi.nlm.nih.gov/geo/query/acc.cgi?acc=GSE58092).

### Statistical analysis

Differences between control and treatment groups were assessed using the 2-tailed unpaired T-test. All statistical analyses were performed using Prism 5 (GraphPad). The mean ± SEM was shown in figures.

### Supplemental information

Supplemental Information includes supplemental methods for antibody array; 6 supplemental tables for clinical demographics of retinoblastoma patients, information about gene expression and miRNA assays, sequences of STAT3 siRNA, information about miRNA inhibitors, and the summary of antibody array and miRNA microarray; and 8 supplemental figures for quantitative analyses of real-time PCR, Western blotting, and immunocytochemistry. We also provided the raw data of antibody array as the Microsoft excel file.

## SUPPLEMENTAL METHODS, FIGURES AND TABLES


