# Supplementary material for: STAT3 inhibition suppresses proliferation of retinoblastoma through down-regulation of positive feedback loop of STAT3/miR-17-92 clusters
Source: Oncotarget. 2014 Nov 3;5(22):11513–25. doi: 10.18632/oncotarget.2546 (PMC4294389; doi:10.18632/oncotarget.2546)
Supplement: Supplementary file 1 [file oncotarget-05-11513-s001.pdf]

SUPPLEMENTAL METHODS, FIGURES AND TABLES

Antibody array

Extracted proteins from cell lysates of HRMECs and Y79 were purified using a gel matrix column in Antibody Array Assay Kit (Full Moon) as the manufacturer's instructions. Then, the protein sample (50 µg) with labeling buffer and 30 µg biotin/dimethylformamide solution was incubated at room temperature for 1 hour with mixing. After that, the sample was treated with stop reagent and incubated at room temperature for 30 minutes with mixing. For antibody array assay, the slide from Cancer Signaling Phospho Antibody Microarray (Full Moon) was treated with blocking solution at room temperature for 1 hour. Then, we treated the slide with the labeled protein sample at room temperature for 2 hours after mixing with 6 mL of coupling solution and washed the slide 6 times (5 minutes each) with washing solution. For detection of protein signal, Cy3-streptavidin (0.5 µg/mL, GE) was treated to the slide at room temperature for 45 minutes. Then, we washed the slide 6 times (5 minutes each) with washing solution. The scanning of the slide was performed using GenePix 4000B scanner (Axon Instrument) at 10 µm resolution. The quantification of the data was performed with GenePix Software (Axon Instrument) and the numeric data were analized with Genowiz™ (Ocimum Biosolutions). The total list of the expression levels of proteins in the array was provided as supplementary material.

Supplementary Table S1. Clinical demographics of 6 retinoblastoma patients.

Patient number	Age at diagnosis (months)	Sex (M/F)	Reese-Ellsworth classification	ICR classification
1	36	M	Va	E
2	3	M	Va	E
3	23	M	Va	E
4	19	F	Va	E
5	21	M	Va	E
6	19	M	Va	E

ICR: international classification of retinoblastoma

Supplementary Table S2. The list of top 5 phosphorylated proteins which demonstrate higher expressions in Y79 cells compared to HRMECs.

Protein name	Phosphorylated residue	Normalized expression in Y79 (Log2)	Normalized expression in HRMEC (Log2)	Fold change (Y79/HRMEC)
BRCA1	Ser1524	10.512	9.427	2.121656
NFκB-p65	Ser529	10.439	9.580	1.813585
STAT3	Ser727	10.135	9.370	1.699652
IkB-α	Ser32/36	12.137	11.588	1.463001
FKHR	Ser256	10.209	9.762	1.362824

HRMEC: human retinal microvascular endothelial cell

Supplementary Table S3. IDs of Gene Expression Assays and miRNA Assays utilized in this study.

Target	ID	Catalog number
**mRNA**		
BCL2	HS00608023_m1	4453320
BCL2L1	Hs00236329_m1	4453320
BIRC5	Hs04194392_s1	4453320
MMP9	Hs00234579_m1	4453320
VEGF	Hs00900055_m1	4453320
CCND1	Hs00765553_m1	4453320
CDKN1A	Hs00355782_m1	4453320
MYC	Hs00153408_m1	4453320
GAPDH	Hs999999905_m1	4453320
18S	Hs99999901_s1	4453320
GUSB	Hs99999908_m1	4453320
HPRT1	Hs99999909_m1	4453320
**miRNA**		
hsa-miR-17-5p	002308	4427975
hsa-miR-18a-5p	002422	4427975
hsa-miR-19a-3p	000395	4427975
hsa-miR-19b-3p	000396	4427975
hsa-miR-20a-5p	000580	4427975
hsa-miR-92a-3p	000431	4427975
RNU48	001006	4427975

Supplementary Table S4. STAT3 siRNAs utilized in this study.

ID in this study	ID from the manufacturer	Sequence
Negative control	SN-1003	CCUACGCCACCAAUUUCGU (sense)<br>ACGAAAUUGGUGGCGUAGG (antisense)
STAT3 siRNA#1	1145658	UGUUCUCUGAGACCCAUGA (sense)<br>UCAUGGGUCUCAGAGAACA (antisense)
STAT3 siRNA#2	1145659	CUCCAACAUCUGUCAGAUG (sense)<br>CAUCUGACAGAUGUUGGAG (antisense)

Supplementary Table S5. miRNA inhibitors utilized in this study.

Target	ID	Catalog number
Negative control	#1	4464076
hsa-miR-17-5p	MH12412	4464085
hsa-miR-18a-5p	MH12973	4464085
hsa-miR-19a-3p	MH10649	4464085
hsa-miR-19b-3p	MH10629	4464085
hsa-miR-20a-5p	MH10057	4464085
hsa-miR-92a-3p	MH10916	4464085

Supplementary Table S6. The list of highly expressed miRNAs in Y79 cells. Components of miR-17-92 clusters are in bold.

Rank	miRNA	Mean raw value
1	hsa-miR-4454	121590.34
2	hsa-miR-4284	11868.97
3	hsa-miR-5100	4046.78
4	hsa-miR-6089	2187.23
5	hsa-miR-1260a	2015.87
6	hsa-miR-1246	1920.81
**7**	**hsa-miR-19b-3p**	**1910.42**
8	hsa-miR-4459	1679.69
9	hsa-miR-4516	1567.77
**10**	**hsa-miR-20a-5p**	**1320.28**
11	hsa-miR-16-5p	1281.72
12	hsa-miR-4286	1136.02
**13**	**hsa-miR-17-5p**	**1074.62**
**14**	**hsa-miR-19a-3p**	**973.44**
**15**	**hsa-miR-92a-3p**	**951.30**
16	hsa-miR-3651	752.63
17	hsa-miR-1973	750.20
18	hsa-miR-630	746.04
19	hsa-miR-15b-5p	732.21
20	hsa-miR-1273g-3p	675.40
**48**	**hsa-miR-18a-5p**	**289.66**

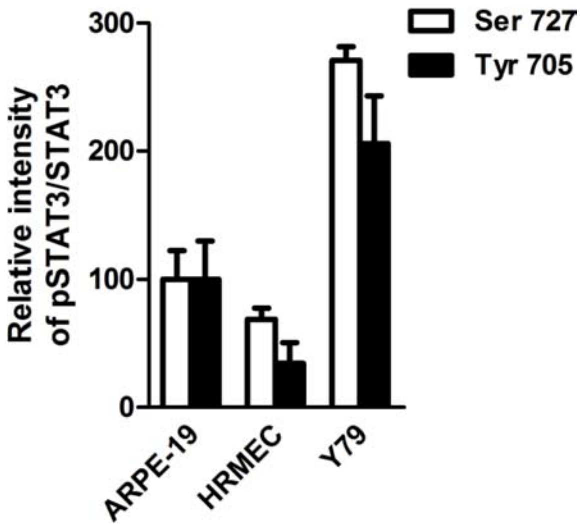

Supplementary Figure S1: Quantitative analysis of the ratio of pSTAT3/STAT3 in Western blotting of whole cell extracts of ARPE-19, HRMECs, and Y79 cells. Data were from 3 independent experiments.

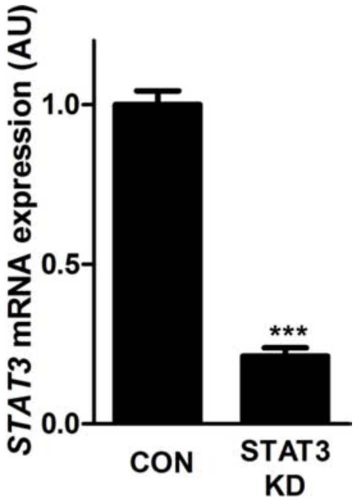

Supplementary Figure S2: Quantitative analysis of relative *STAT3* mRNA expression according to the treatment with scramble siRNA (CON) or STAT3 targeting siRNA (STAT3 KD), which is designated as STAT3 siRNA#1 in Supplementary Table 4. ***$P < 0.001$.

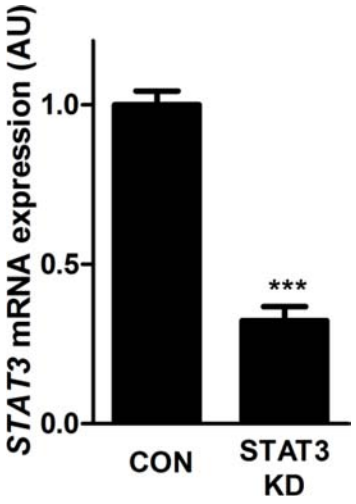

Supplementary Figure S3: Quantitative analysis of relative *STAT3* mRNA expression according to the treatment with scramble siRNA (CON) or STAT3 targeting siRNA (STAT3 KD), which is designated as STAT3 siRNA#2 in Supplementary Table 4. ***$P < 0.001$.

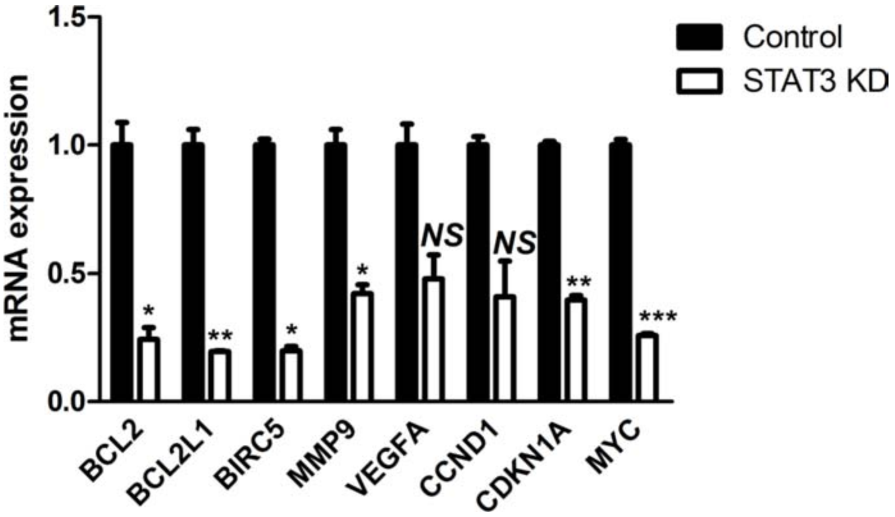

Supplementary Figure S4: Quantitative analysis of relative mRNA expression of target genes of STAT3 according to the treatment with scramble siRNA (Control) and STAT3 siRNA (STAT3 KD), which is designated as STAT3 siRNA#2 in Supplementary Table 4. *NS*, not significant ($P > 0.05$); *$P < 0.05$; **$P < 0.01$; ***$P < 0.001$.

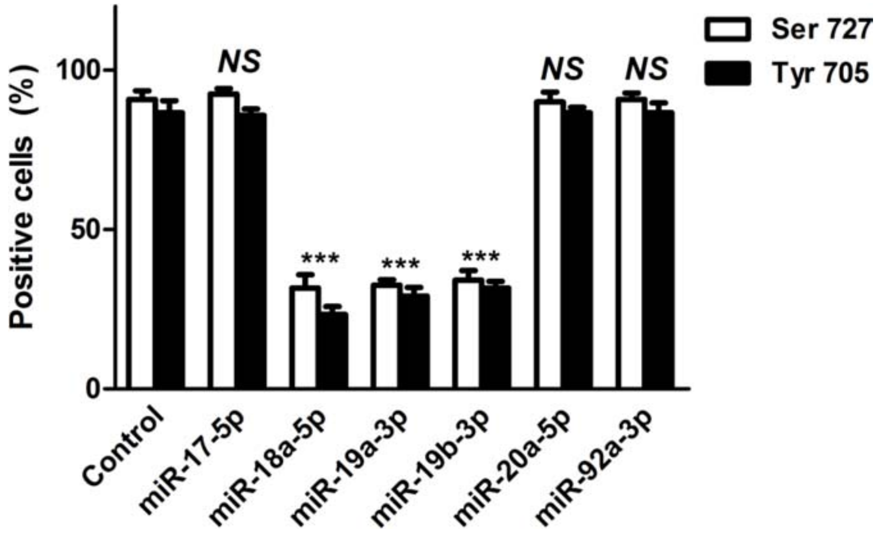

Supplementary Figure S5: Quantitative analysis of immunocytochemical staining of pSTAT3^{Ser727} and pSTAT3^{Tyr705} of Y79 cells according to the treatment with scramble inhibitor (Control) or specific inhibitors to miRNAs of miR-17-92 clusters. *NS*, not significant ($P > 0.05$); ***$P < 0.001$.

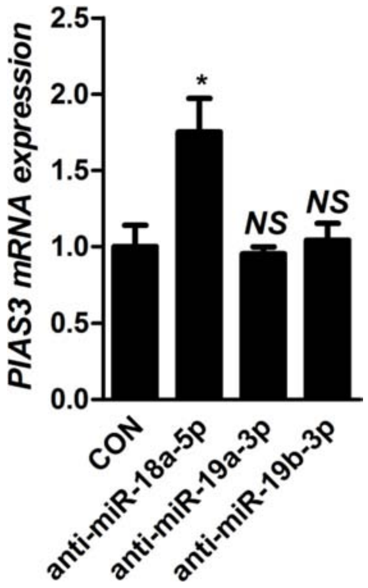

Supplementary Figure S6: Quantitative analysis of the relative levels of the expression of *PIAS3* according to the treatment with scramble inhibitor (Control) or specific inhibitors to miRNAs of miR-17-92 clusters. *NS*, not significant ($P > 0.05$); *$P < 0.05$.

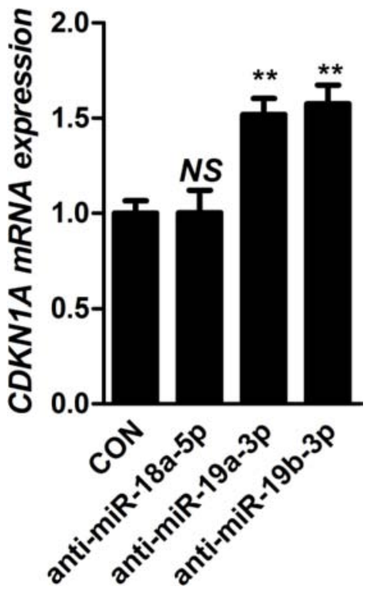

Supplementary Figure S7: Quantitative analysis of the relative levels of the expression of *SOCS1* according to the treatment with scramble inhibitor (Control) or specific inhibitors to miRNAs of miR-17-92 clusters. *NS*, not significant ($P > 0.05$); $^{**}P < 0.01$.

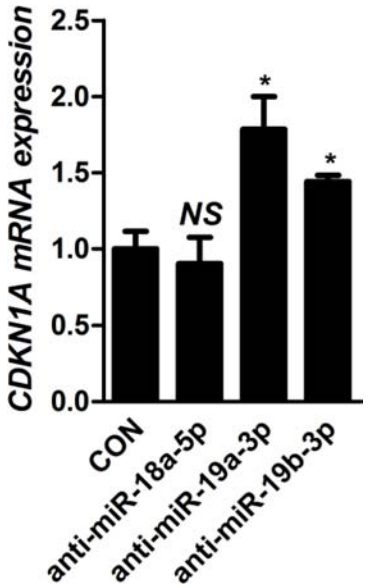

Supplementary Figure S8: Quantitative analysis of the relative levels of the expression of *SOCS3* according to the treatment with scramble inhibitor (Control) or specific inhibitors to miRNAs of miR-17-92 clusters. *NS*, not significant ($P > 0.05$); $*P < 0.05$.
